# HNRNPD interacts with ZHX2 regulating the vasculogenic mimicry formation of glioma cells via linc00707/miR-651-3p/SP2 axis

**DOI:** 10.1038/s41419-021-03432-1

**Published:** 2021-02-04

**Authors:** Sifei Yu, Xuelei Ruan, Xiaobai Liu, Fangfang Zhang, Di Wang, Yunhui Liu, Chunqing Yang, Lianqi Shao, Qianshuo Liu, Lu Zhu, Yang Lin, Yixue Xue

**Affiliations:** 1 Department of Neurobiology, School of Life Sciences, China Medical University, Shenyang, 110122 People’s Republic of China; 2 Key Laboratory of Cell Biology, Ministry of Public Health of China, China Medical University, Shenyang, 110122 People’s Republic of China; 3 Key Laboratory of Medical Cell Biology, Ministry of Education of China, China Medical University, Shenyang, 110122 People’s Republic of China; 4 Department of Neurosurgery, Shengjing Hospital of China Medical University, Shenyang, 110004 People’s Republic of China; 5 Liaoning Research Center for Translational Medicine in Nervous System Disease, Shenyang, 110004 People’s Republic of China; 6 Key Laboratory of Neuro-oncology in Liaoning Province, Shenyang, 110004 People’s Republic of China

**Keywords:** Cancer, Neuroscience

## Abstract

Studies have found that RNA-binding proteins (RBPs) are dysfunctional and play a significant regulatory role in the development of glioma. Based on The Cancer Genome Atlas database and the previous studies, we selected heterogeneous nuclear ribonucleoprotein (HNRNPD) as the research candidate and sought its downstream targeted genes. In the present study, HNRNPD, linc00707, and specific protein 2 (SP2) were highly expressed, while zinc fingers and homeboxes 2 (ZHX2) and miR-651-3p were remarkedly downregulated in glioma tissues and cells. HNRNPD, linc00707, and SP2 knockdown or ZHX2 and miR-651-3p overexpression suppressed glioma cells proliferation, migration, and invasion and vasculogenic mimicry (VM) formation. Knockdown of HNRNPD increased the stability of ZHX2 mRNA. ZHX2 bound to the promoter region of linc00707 and negatively regulate its expression. Linc00707 could bind with miR-651-3p, while miR-651-3p bound to the 3′ untranslated region (3′UTR) of SP2 mRNA to negatively regulate its expression. The transcription factor SP2 directly bound to the promoter regions of the VM formation-related proteins MMP2, MMP9, and VE-cadherin, playing a role in promoting transcription in order to regulate the VM formation ability of glioma cells.

## Introduction

Glioma is one of the most common malignant tumors which grows invasively in central nervous system. The median survival time of patients with high-grade glioma is <15 months^1^. Vasculogenic mimicry (VM) is a tubular structure formed by tumor cells which can provide nutrition, involving in regulating the occurrence and development of tumors^2^. VM exists in a variety of malignant tumors and promotes their malignant progression^3,4^. In recent years, antiangiogenic therapy has become a new treatment for glioma. However, due to the existence of VM, antiangiogenic therapy has been greatly restricted^5^. Therefore, exploring the mechanisms that inhibit VM formation may bring light to the treatment of glioma.

RNA-binding proteins (RBPs) could target RNAs to regulate the gene expression via forming RNA–protein complexes, which play an important role in biological program, including RNA synthesis, alternative splicing, modification, and translation^6^. Heterogeneous nuclear ribonucleoprotein (HNRNPD) is localized in nucleus and can form complexes with heterogeneous nuclear RNAs, involving in the formation of precursor mRNAs and the metabolism and transport of mRNAs. Studies have shown that HNRNPD acts as an oncogene in hepatocellular carcinoma and osteosarcoma^7,8^. HNRNPA2/B1 is highly expressed in glioma tissues and cells, whose expression increases with the pathological grade of the glioma tissues^9^.

The transcription factor zinc fingers and homeboxes 2 (ZHX2) is located on chromosome 8 and is ubiquitously expressed in nucleus. It is a classic transcriptional repressor, which contains two C2H2 zinc finger structures and five homeobox DNA binding domains. The expression of ZHX2 is tissue-specific. It expressed at low levels in hepatocellular carcinoma tissues and inhibits tumor growth^10^. Moreover, it plays an inhibitory role in nonalcoholic fatty liver-induced hepatocellular carcinoma^11^. However, studies have also shown that ZHX2 is highly expressed in renal cell carcinoma cells, knockdown of ZHX2 inhibits the proliferation of renal cell carcinoma cells^12^.

Long noncoding RNA (lncRNA) is a type of RNA with a length of >200 bp without protein-coding function, which could regulate the transcription, translation, and stability of the protein-coding genes^13,14^. Linc00707 is located on chromosome 10, with a length of ~3 kb. It acts as an oncogenic factor in tumors, including gastric cancer, breast cancer, and clear cell renal cell carcinoma^15–17^. miRNA is a type of noncoding RNA with regulatory functions in eukaryotes. It is ~20–25 nt in length and can specifically bind to the 3′ untranslated region (3′UTR) of the target genes in order to regulate their expressions^18^. Studies have shown that miR-651-3p negatively regulates TIAM1, which is highly expressed in gastric cancer cells^19^.

Specific protein 2 (SP2) is located on chromosome 17 and belongs to the SP transcription factors family. It is mainly located in subnuclear foci associated with nuclear matrix, and a transactivation domain is presented at its amino terminus, which can activate or inhibit different promoters^20^. SP2 is highly expressed in gastric cancer tissues, knockdown of SP2 significantly inhibits the proliferation of gastric cancer cells^21^. Moreover, the abundance of SP2 in mice increases with the malignant development of mouse squamous cell carcinoma^22^.

Both MMP2 and MMP9 belong to the matrix metalloproteinases (MMP) family, and play an important role in the invasion and metastasis of tumor cells. They are classic VM formation markers and can promote tumor cells VM formation through a variety of mechanisms^23,24^. In glioma cells, high expressions of MMP2 and MMP9 suggest an enhanced VM formation ability^25^. VE-cadherin is one of the most important signs of VM formation in glioma cells, high expression of VE-cadherin is positively correlated with poor prognosis in glioma patients^26,27^.

In this study, we first identified the endogenous expressions of HNRNPD, ZHX2, linc00707, miR-651-3p, and SP2 in glioma tissues and cells. Further studies focused on the above-mentioned intermolecular regulation and their effects on VM formation ability of glioma cells. Our study aims to provide a new mechanism for VM formation in glioma and a new target for antiangiogenic therapy.

## Methods

### Human tissue samples

Human glioma specimens and normal brain tissues (NBTs) were obtained from the Department of Neurosurgery at ShengJing Hospital of China Medical University. The research was confirmed by the Ethics Committee of ShengJing Hospital, with informed consent obtained from all patients included. All specimens were validly frozen and preserved in liquid nitrogen after surgical resection.

### Cell culture

Human glioma cell lines U87 and U251 together with human embryonic kidney (HEK)-293T cells were purchased from Chinese Academy of Medical Sciences. They were cultured in Dulbecco’s modified Eagle high glucose supplemented with 10% fetal bovine serum. All cells were maintained in humidified incubator at 37 °C with 5% CO_2_. The human astrocytes (HA) cell lines were purchased from the ScienCell Research Laboratories and cultured under the manufacturer’s instructions.

### RNA extraction and quantitative real-time PCR

Trizol reagent (Life Technologies Corporation, Calsbad, CA) was used to extract RNA from tissues and cells. With the application of 7500 rapid RT-PCR system and the one-step SYBR Prime-Script RT-PCR kit (Takara Bio, Inc, Japan), we detected the expression levels of ZHX2, linc00707, and SP2. TaqMan MicroRNA reverse transcription kit (Applied Biosystems, Foster City, CA, USA) was used for reverse transcription of miR-651-3p, while TaqMan Universal Master Mix II was used to detect its endogenous expression. GAPDH and U6 were used as endogenous control. Primers used are shown in Table S1.

### Cell transfection

Short hairpin HNRNPD (HNRNPD(−)), linc00707 (linc00707(−)), and SP2 (SP2(−)) plasmids together with miR-651-3p agomir (pre-miR-651-3p), antagomir (anti-miR-651-3p), as well as ZHX2 and SP2 full length (ZHX2(+), SP2(+)) plasmid and their corresponding nontargeting sequence (negative control, NC) were synthesized (GenePharma, Shanghai, China). Cells were seeded into 24-well plates and transfected with Lipofectamine 3000 reagent and Opti-MEM I (Life Technologies Corporation, Carlsbad, CA) under the manufacturer’s instructions. Stably transfected cells were selected through Geneticin (G418; Sigma-Aldrich, St Louis, MO, USA). Sequences of the small hairpin RNA template are shown in Table S2.

### Cell proliferation assay

Cells were seeded into 96-well plates at an appropriate density. After 48 h in the incubator, we used the Cell Counting Kit-8 (CCK-8, Dojin, Japan) to detect the proliferation ability of the cells. The Spectramax M5 microplate reader was used to measure the absorbance at the wavelength of 450 nm.

### Cell migration and invasion assay

After diluting the cells with serum-free culture medium, the cell suspension was evenly spreaded in the upper chamber of the 24-well plate (#3422 Costar, Coring, NY, USA) with each chamber around 2 × 10^4^ cells. After 36 h in the incubator, we fixed and stained the cells, then observed under the microscope, selected three to five fields randomly and assessed the migration ability of glioma cells. As for the invasion ability, 50 μl Matrigel was spreaded on the upper chamber of the transwell plate. The remained steps are the same as above.

### In vitro three-dimensional tube formation assay

Each well of the 96-well plate was covered with 90 μl Matrigel. The cells were resuspended in the serum-free medium and inoculated on the surface of Matrigel. The vascular structures of the cells were observed and photographed under the microscope at suitable time nodes.

### Western blot analysis

The cells were lysed with radioimmunoprecipitation assay buffer (RIPA buffer) and centrifuged to extract their proteins. The proteins were transferred to the polyvinylidene fluoride membrane after electrophoresis. The membrane was blocked with Tween-Tris-buffered saline containing 5% skimmed milk, then incubated with the primary antibody at 4 °C overnight. Later the membrane was inoculated with the horseradish peroxidase-conjugated secondary antibody. Finally, the membrane was scanned with Enhanced Development Chemiluminescence kit and ChemImager 5500 V2.03 software (Alpha Innotech, San Leandro,CA).

### Reporter vector constructs and luciferase reporter assay

We amplified the sequence of linc00707 and SP2 and their predicted binding sites with miR-651-3p, as well as their mutant sequences by PCR, then cloned them together into the pmirGLO dual-luciferase miRNA target expression vector (Promega, Madison, WI, USA). HEK-293T cells were co-transfected with the control and mutant plasmids. The dual-lucifer reporter assay system was used to detect the luciferase activity.

### Chromatin immunprecipitation assay

The experiment was performed with the Simple ChIP Enzymatic Chromatin IP kit (Cell Signaling Technology, Danvers, Massachusetts, USA). The cells were cross-linked with 1% formaldehyde, and quenched with glycine. Then the cells were collected in lysis buffer. With 2% lysate as control, other lysates were incubated with normal rabbit IgG antibodies or corresponding antibodies, DNA was cross-linked and purified by 5 mol/l NaCl and proteinase K. Primers used are shown in Table S3.

### RNA immunprecipitation assay

The Magna RNA-Binding Protein Immunoprecipitation Kit (Millipore, Bedford, MA) was used for RIP experiments. Cell lysates were incubated with RIP buffer containing magnetic beads conjugated with human anti-Ago2 antibody or normal mouse IgG. The purified RNA was analyzed by quantitative real-time PCR (qRT-PCR) to certify the existence of the potential target.

### Nascent RNA capture assay

The Click-iT Nascent RNA Capture Kit (Invitrogen) was used to measure the existence of nascent RNA. The nascent RNA was labeled with 0.2 mM 5-ethynyluridine and captured on magnetic beads for subsequent qRT-PCR.

### RNA stability measurement

A total of 5 μg/ml actinomycin D was added to the cell culture medium to prevent denovo RNA synthesis. Total RNA was extracted at setting times, whose expression was detected by qRT-PCR.

### Human microarray analysis

The transcription factor, lncRNA and miRNA microarray analysis, sample preparation, and microarray hybridization were operated by Kangchen Biotechnology Corporation (Shanghai, China).

### Immunohistochemistry

The formalin-fixed and paraffin-embedded tissue sections were dewaxed in xylene, hydrated in gradient ethanol, and boiled in antigen-unmasking solution. The sections were then incubated with peroxide after cooling down, blocked with goat serum, and incubated with HNRNPD and CD34 primary antibody (1:50, Proteintech) at 4 °C overnight. After incubation with secondary antibody, the specimens were stained with DAB kit (MaiXin Biotech, China). Periodic acid solution, schiff solution, and hematoxylin were used for the next PAS staining. The classification method according to the positive proportion of staining: 0 points (negative), 1 point (1–25%), 2 points (26–50%), 3 points (51–75%), 4 points (76–100%).

### Tumor xenografts in nude mice

The nude mice were divided into five groups in the in vivo experiments. Four-week-old athymic nude mice were purchased from the Chinese Academy of Sciences. Each nude mouse was injected ~6 × 10^5^ cells subcutaneously in the right armpit, measured every 5 days and calculated the tumor size according to the formula: volume (mm^3^) = length × width^2^/2. The nude mice were sacrificed 45 days after intracranial injection and the transplanted tumors were then removed.

### Statistical analysis

The average value of the experimental data was represented as mean ± SD. All differences were analyzed by SPSS 22.0 statistical software and *t* test or one-way analysis of variance. *P* < 0.05 was considered as statistically significant.

## Results

### HNRNPD was upregulated in glioma tissues and cells, knockdown of HNRNPD significantly inhibited glioma VM formation

**Fig. 1:**
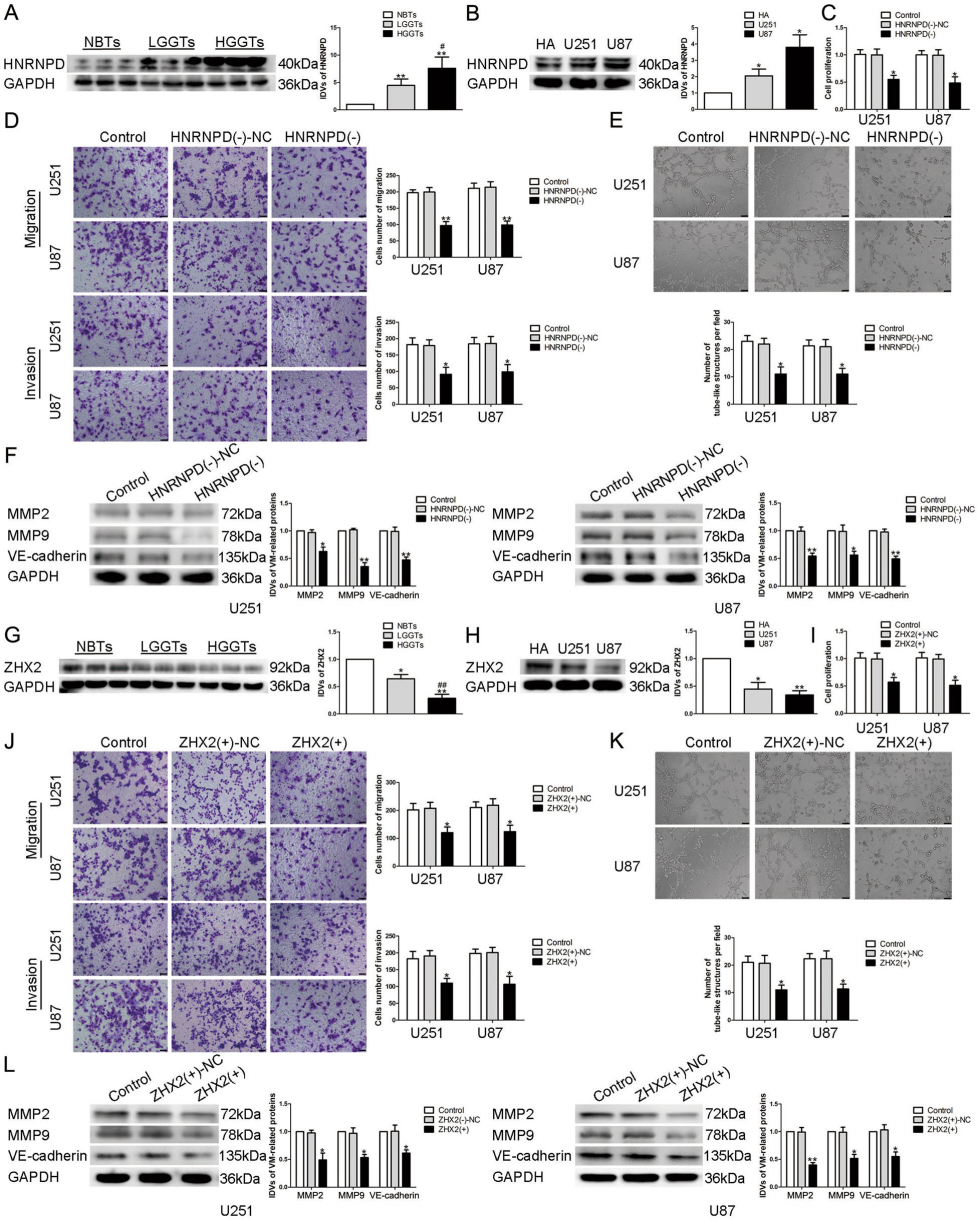
The expression and effect of HNRNPD and ZHX2 on VM formation ability of glioma cells. **A** Expression levels of HNRNPD in glioma tissues by western blot. Data are presented as the mean ± SD (*n* = 9, each group). **P < 0.01 vs. NBTs group; #*P* < 0.05 vs. LGGTs group. **B** Expression levels of HNRNPD in glioma cells by western blot. Data are presented as the mean ± SD (*n* = 3, each group). *P < 0.05 vs. HA group. **C**–**E** CCK-8 assay, transwell, and three-dimensional culture were applied to evaluate the proliferation, migration, invasion, and tube formation effect of HNRNPD on U87 and U251 cells. Representative images and accompanying statistical plots were presented. The scale bar represents 50 µm. **F** Protein levels of MMP2, MMP9, and VE-cadherin regulated by HNRNPD in U87 and U251 cells. Representative protein expressions and corresponding IDVs of MMP2, MMP9, and VE-cadherin in U87 and U251 are shown. Data are presented as the mean ± SD (*n* = 3, each group). *P < 0.05, **P < 0.01 vs. HNRNPD(−)-NC group. **G** Expression levels of ZHX2 in glioma tissues. Data are presented as the mean ± SD (*n* = 9, each group). *P < 0.05, **P < 0.01 vs. NBTs group; ##*P* < 0.01 vs. LGGTs group. **H** Expression levels of ZHX2 in glioma cells. Data are presented as the mean ± SD (*n* = 3, each group). *P < 0.05, **P < 0.01 vs. HA group. **I**–**K** CCK-8 assay, transwell, and three-dimensional culture were applied to evaluate the proliferation, migration, invasion, and tube formation effect of ZHX2 on U87 and U251 cells. Representative images and accompanying statistical plots were presented. The scale bar represents 50 µm. **L** Protein levels of MMP2, MMP9, and VE-cadherin regulated by ZHX2 in U87 and U251 cells. Representative protein expressions and corresponding IDVs of MMP2, MMP9, and VE-cadherin in U87 and U251 are shown. Data are presented as the mean ± SD (*n* = 3, each group). *P < 0.05, **P < 0.01 vs. ZHX2(+)-NC group.

The endogenous expression of HNRNPD was detected by western blot. As shown in Fig. 1A, compared with NBTs, the expression of HNRNPD in glioma tissues significantly increased with the pathological grade. Moreover, compared with HA, HNRNPD is significantly overexpressed in U87 and U251 cells (Fig. 1B). Stably knockdown of HNRNPD plasmid was constructed to assess the role of HNRNPD. As shown in Fig. 1C–E, CCK-8 assay, transwell, and tube formation assay were used to detect the changes of the biological functions in U87 and U251 cells. We found that compared with HNRNPD(−)-NC group, the proliferation, migration, invasion, and VM formation ability of glioma cells in HNRNPD(−) group were significantly reduced. Further, western blot was used to detect the changes of the expression of VM formation-related proteins MMP2, MMP9, and VE-cadherin in glioma cells after HNRNPD knockdown, we found that compared with HNRNPD(−)-NC group, the expression of the proteins decreased significantly in HNRNPD(−) group (Fig. 1F).

### HNRNPD regulated the VM formation ability of glioma cells by decreasing the stability of ZHX2 mRNA

**Fig. 2:**
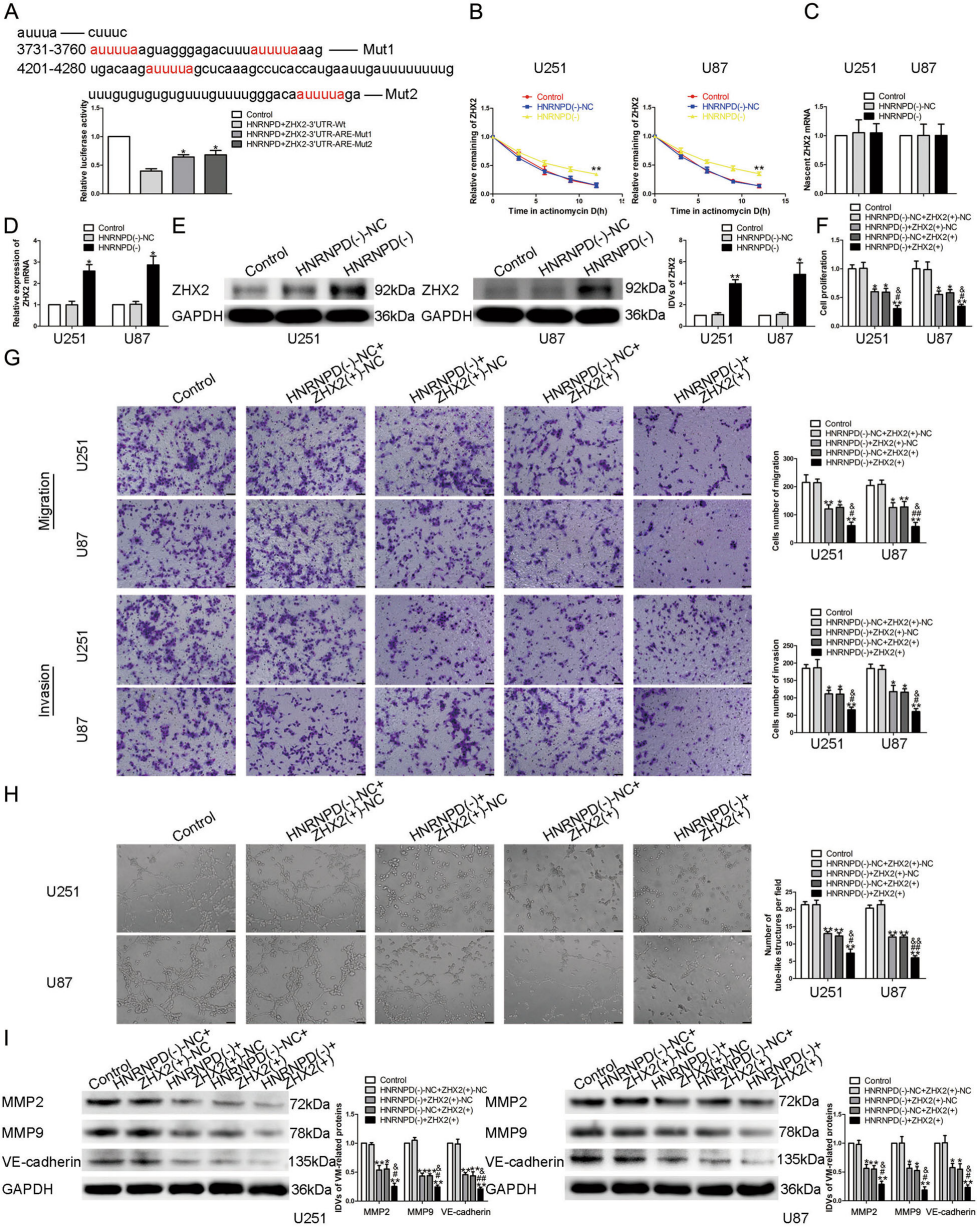
HNRNPD bound to ZHX2 and promoted the ability of VM formation in glioma cells. **A** Luciferase reporter assay of HEK-293T cells co-transfected with HNRNPD and ZHX2-3′UTR-Wt or ZHX2-3′UTR-Mut at different locus. Data are presented as the mean ± SD (*n* = 3, each group). *P < 0.05 vs. HNRNPD + ZHX2-3′UTR-Wt group. **B** The graph shows ZHX2 mRNA levels at different times treated by ActD in U87 and U251 cells. Data are presented as mean ± SD (*n* = 3, each group). **P < 0.01 vs. HNRNPD(−)-NC group. **C** The graph shows nascent ZHX2 in U87 and U251 cells. Data are presented as mean ± SD (*n* = 3, each group). *P* > 0.05 vs. HNRNPD(−)-NC group. **D** Expression levels of ZHX2 mRNA regulated by HNRNPD in U87 and U251 cells. Data are presented as the mean ± SD (*n* = 3, each group). *P < 0.05 vs. HNRNPD(−)-NC group. **E** Expression levels of ZHX2 protein regulated by HNRNPD in U87 and U251 cells. Data are presented as the mean ± SD (*n* = 3, each group). *P < 0.05, **P < 0.01 vs. HNRNPD(−)-NC group. **F**–**H** CCK-8 assay, transwell, and three-dimensional culture were applied to evaluate the proliferation,migration, invasion, and tube formation effect of HNRNPD inhibition and ZHX2 overexpression on U87 and U251 cells. Representative images and accompanying statistical plots were presented. The scale bar represents 50 µm. **I** Protein levels of MMP2, MMP9, and VE-cadherin regulated by HNRNPD and ZHX2 in U87 and U251 cells. Data are presented as the mean ± SD (*n* = 3, each group). *P < 0.05, **P < 0.01 vs. HNRNPD(−)-NC + ZHX2(+)-NC group; #*P* < 0.05, ##*P* < 0.01 vs. HNRNPD(−) + ZHX2(+)-NC group; &*P* < 0.05, &&*P* < 0.01 vs. HNRNPD(−)-NC + ZHX2(+) group.

Based on the microarray analysis and the bioinformatics database AREsite2, we found that the expression of ZHX2 could be affected by HNRNPD (Supplementary Fig. 3A). At the same time, ZHX2 mRNA’s 3′UTR contains AU elements, so we consider that there may exist combinations between HNRNPD and ZHX2. First, we applied western blot to evaluate the expression of ZHX2, the results showed that ZHX2 is significantly downregulated in glioma tissues and cells, and the expression in high-grade glioma tissue is lower than that in the low one (Fig. 1G, H). Stably overexpression of ZHX2 plasmid was constructed and transfected into U87 and U251 cells. As shown in Fig. 1I–K, the proliferation, migration, invasion, and VM formation ability of glioma cells in ZHX2(+) group were significantly lower than those in ZHX2(+)-NC group. Western blot results showed that the expression levels of MMP2, MMP9, and VE-cadherin in ZHX2(+) group were also significantly decreased (Fig. 1L). Further study focused on the possibly exist binding effect between HNRNPD and ZHX2. According to the dual-luciferase reporter assay, it is indicated that the mutant AU elements in the ZHX2 mRNA 3′UTR could significantly increase the relative luciferase activity (Fig. 2A). In cells treated with actinomycin D, the half-life of ZHX2 mRNA was significantly prolonged after knockdown of HNRNPD (Fig. 2B), without discovering changes in the expression of nascent RNA (Fig. 2C). qRT-PCR and western blot assay found that the expression of ZHX2 mRNA and protein increased significantly in HNRNPD(−) group (Fig. 2D, E). We transfected overexpression plasmid of ZHX2 into U87 and U251 cells, which have stably knockdown HNRNPD. The proliferation, migration, invasion, and VM formation ability of glioma cells in HNRNPD(−) + ZHX2(+)-NC group, HNRNPD(–)-NC + ZHX2(+) group, and HNRNPD(−) + ZHX2(+) group were significantly decreased, among which the HNRNPD(−) + ZHX2(+) group was the most significant (Fig. 2F–H). Western blot results of MMP2, MMP9, and VE-cadherin expression showed the same as above (Fig. 2I). These results indicated that HNRNPD regulates the VM formation ability of glioma cells by decreasing the stability of ZHX2 mRNA.

### Linc00707 was highly expressed in glioma tissues and cells, promoted the ability of VM formation

**Fig. 3:**
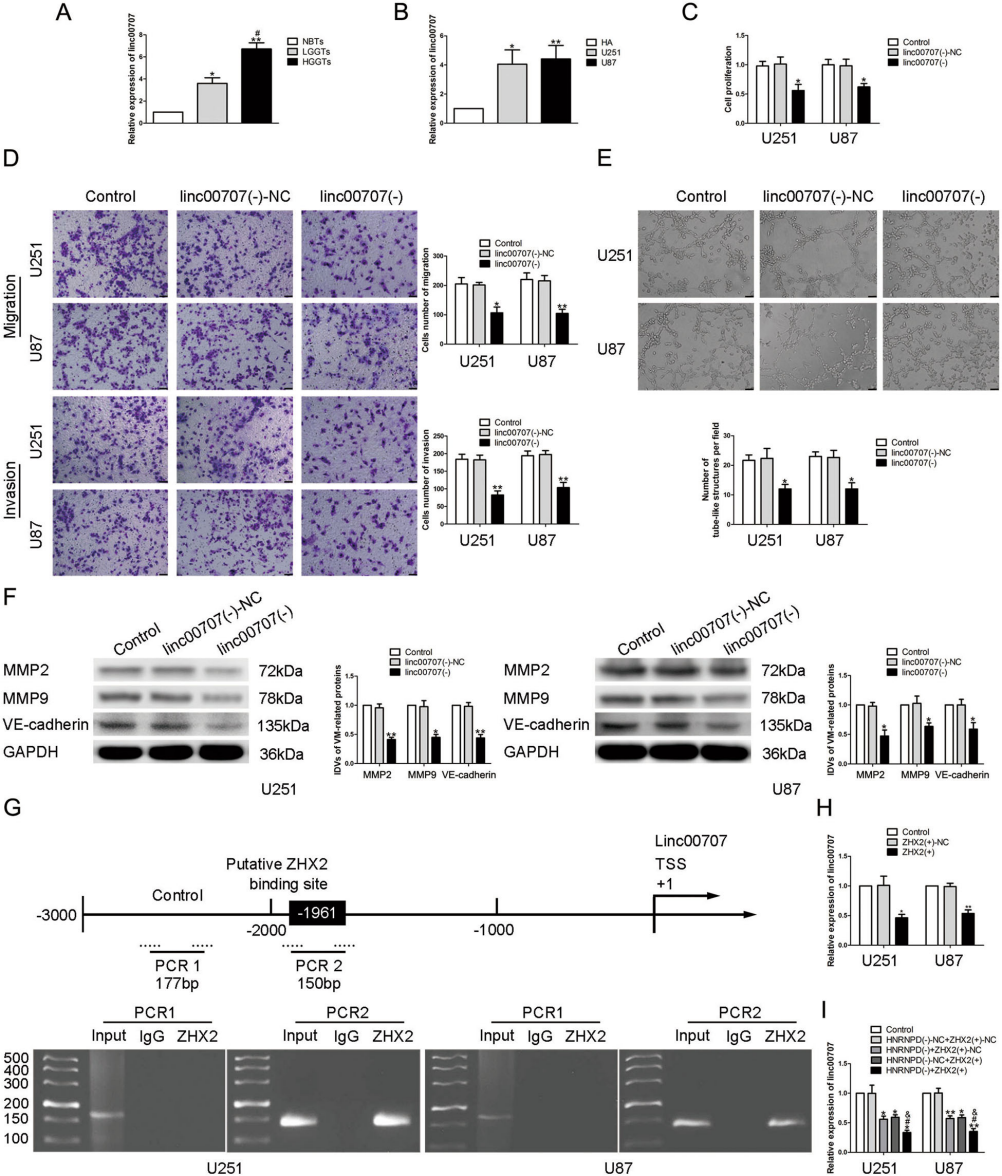
ZHX2 targeted and negatively regulated linc00707. **A** Expression levels of linc00707 in glioma tissues. Data are presented as the mean ± SD (*n* = 9, each group). *P < 0.05, **P < 0.01 vs. NBTs group; #*P* < 0.05 vs. LGGTs group. **B** Expression levels of linc00707 in glioma cells. Data are presented as the mean ± SD (*n* = 3, each group). *P < 0.05, **P < 0.01 vs. HA group. **C**–**E** CCK-8 assay, transwell, and three-dimensional culture were applied to evaluate the proliferation, migration, invasion, and tube formation effect of linc00707 on U87 and U251 cells. Representative images and corresponding statistical plots are shown. The scale bar represents 50 µm. **F** Protein levels of MMP2, MMP9, and VE-cadherin regulated by linc00707 in U87 and U251 cells. Data are presented as the mean ± SD (*n* = 3, each group). *P < 0.05,**P < 0.01 vs. linc00707(−)-NC group. **G** Schematic representation of linc00707 promoter region in 3000 bp upstream of the transcription start site (TSS; designated as +1). PCR products for putative linc00707 binding sites and an upstream region not expected to associate with ZHX2 were depicted with bold lines. Dashed lines represent the primers used for each PCR. The image was representative of independent ChIP experiments. **H** Expression levels of linc00707 regulated by ZHX2 in U87 and U251 cells. Data are presented as the mean ± SD (*n* = 3, each group). *P < 0.05, **P < 0.01 vs. ZHX2(+)-NC group. **I** RT-qPCR analysis for HNRNPD and ZHX2 regulating linc00707 expression in U87 and U251 cells. Data are presented as the mean ± SD (*n* = 3, each group). *P < 0.05, **P < 0.01 vs. HNRNPD(−)-NC + ZHX2(+)-NC group; #*P* < 0.05 vs. HNRNPD(−) + ZHX2(+)-NC group; &*P* < 0.05 vs. HNRNPD(−)-NC + ZHX2(+) group.

According to the lncRNA microarray analysis together with the database RPISeq and Jaspar, we discovered that the expression of linc00707 could be affected by HNRNPD and there maybe a potential binding site between the promoter region of linc00707 and ZHX2. qRT-PCR was used to detect the expression of linc00707. Compared with NBTs and HA, linc00707 was significantly overexpressed in glioma tissues and cells (Fig. 3A, B). In order to clarify the effect of linc00707, U87 and U251 cells transfected with linc00707 stably knockdown plasmid were constructed. The results showed that compared with linc00707(−)-NC group, the proliferation, migration, invasion, and VM formation ability of glioma cells in linc00707(−) group were significantly reduced (Fig. 3C–E). Western blot experiments showed that the expressions of the VM formation-related proteins in linc00707(−) group were significantly reduced (Fig. 3F). The combination between linc00707 and ZHX2 was confirmed by chromatin immunprecipitation (ChIP) experiment (Fig. 3G). Further, we applid qRT-PCR and discovered that the expression of linc00707 in ZHX2(+) group was significantly decreased compared with ZHX2(+)-NC group (Fig. 3H). In addition, compared with HNRNPD(−) + ZHX2(+)-NC and HNRNPD(−)-NC + ZHX2(+) group, the expression of linc00707 in HNRNPD(−) + ZHX2(+) group was significantly reduced (Fig. 3I).

### miR-651-3p was underexpressed in glioma tissues and cells, inhibited the ability of VM formation

**Fig. 4:**
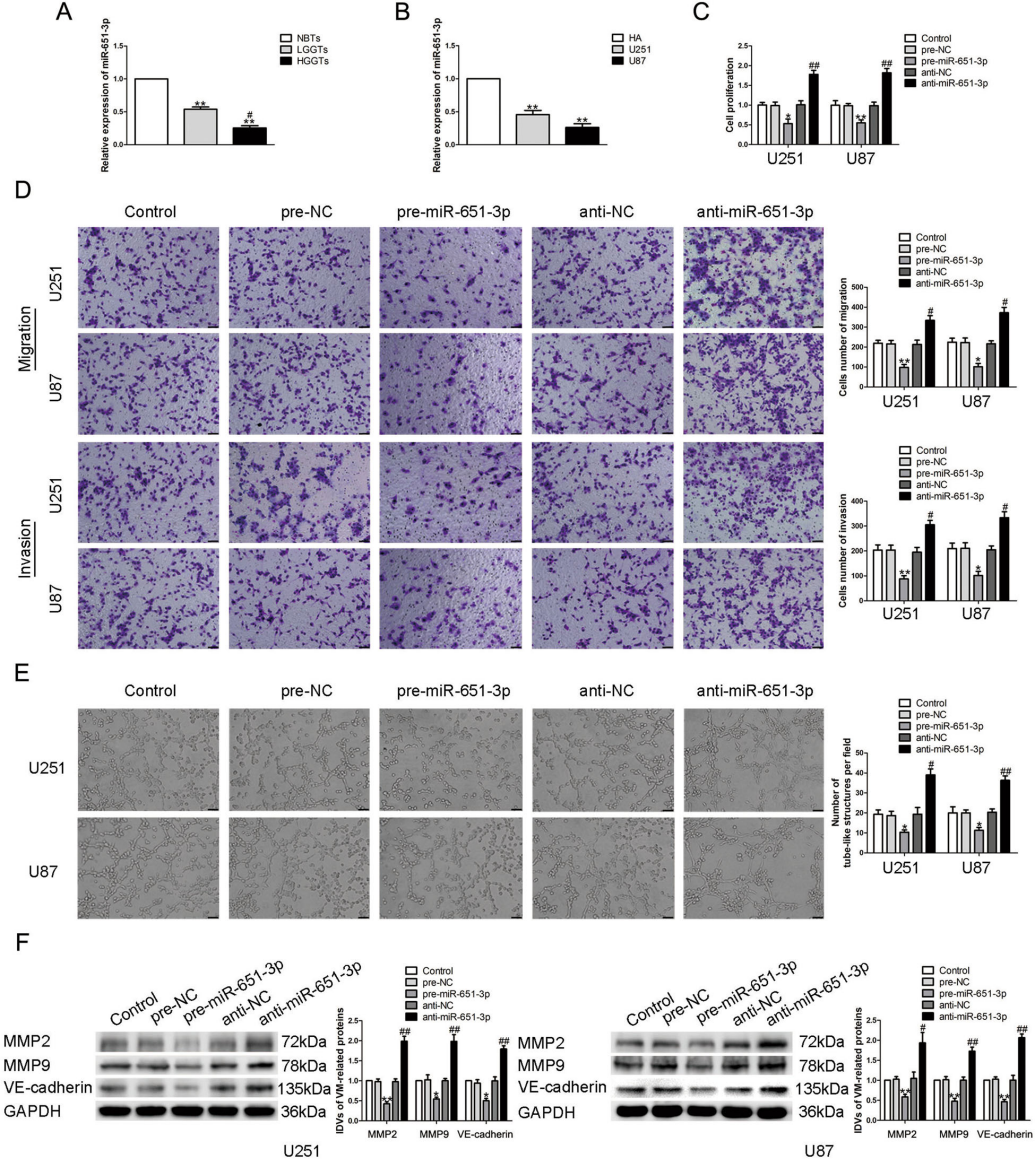
miR-651-3p endogenous expression and the effect on VM formation in glioma cells. **A** Expression levels of miR-651-3p in glioma tissues. Data are presented as the mean ± SD (*n* = 9, each group). **P < 0.01 vs. NBTs group; #*P* < 0.05 vs. LGGTs group. **B** Expression levels of miR-651-3p in glioma cells. Data are presented as the mean ± SD (*n* = 3, each group). **P < 0.01 vs. HA group. **C**–**E** CCK-8 assay, transwell, and three-dimensional culture were applied to evaluate the proliferation, migration, invasion, and tube formation effect of miR-651-3p on U87 and U251 cells. Representative images and corresponding statistical plots are shown. The scale bar represents 50 µm. **F** Protein levels of MMP2, MMP9, and VE-cadherin regulated by miR-651-3p in U87 and U251 cells. Data are presented as the mean ± SD (*n* = 3, each group). *P < 0.05, **P < 0.01 vs. pre-NC group; #*P* < 0.05, ##*P* < 0.01 vs. anti-NC group.

With the help of the bioinformatics database DIANA, we found that there may be a binding site between miR-651-3p and the 3′UTR of linc00707. qRT-PCR showed that compared with NBTs and HA, the expression of miR-651-3p in glioma tissues and cells were significantly reduced, and the expression in high-grade glioma tissues was significantly lower than the low one (Fig. 4A, B). U87 and U251 cells were transfected with overexpressed and underexpressed of miR-651-3p plasmids, respectively, their effects on glioma cells were detected. The results showed that compared with pre-NC group, the proliferation, migration, invasion, and VM formation ability of glioma cells in pre-miR-651-3p group were significantly reduced. Meanwhile, compared with anti-NC group, these abilities of glioma cells in anti-miR-651-3p group were significantly increased (Fig. 4C–E). Western blot was used to detect the changes of the expressions of VM-associated proteins in glioma cells, we found that the expression of MMP2, MMP9, and VE-cadherin were significantly reduced after overexpression of miR-651-3p and the anti-miR-651-3p group showed an oppisite effect (Fig. 4F).

### Linc00707 targeted and negatively regulate the expression of miR-651-3p

**Fig. 5:**
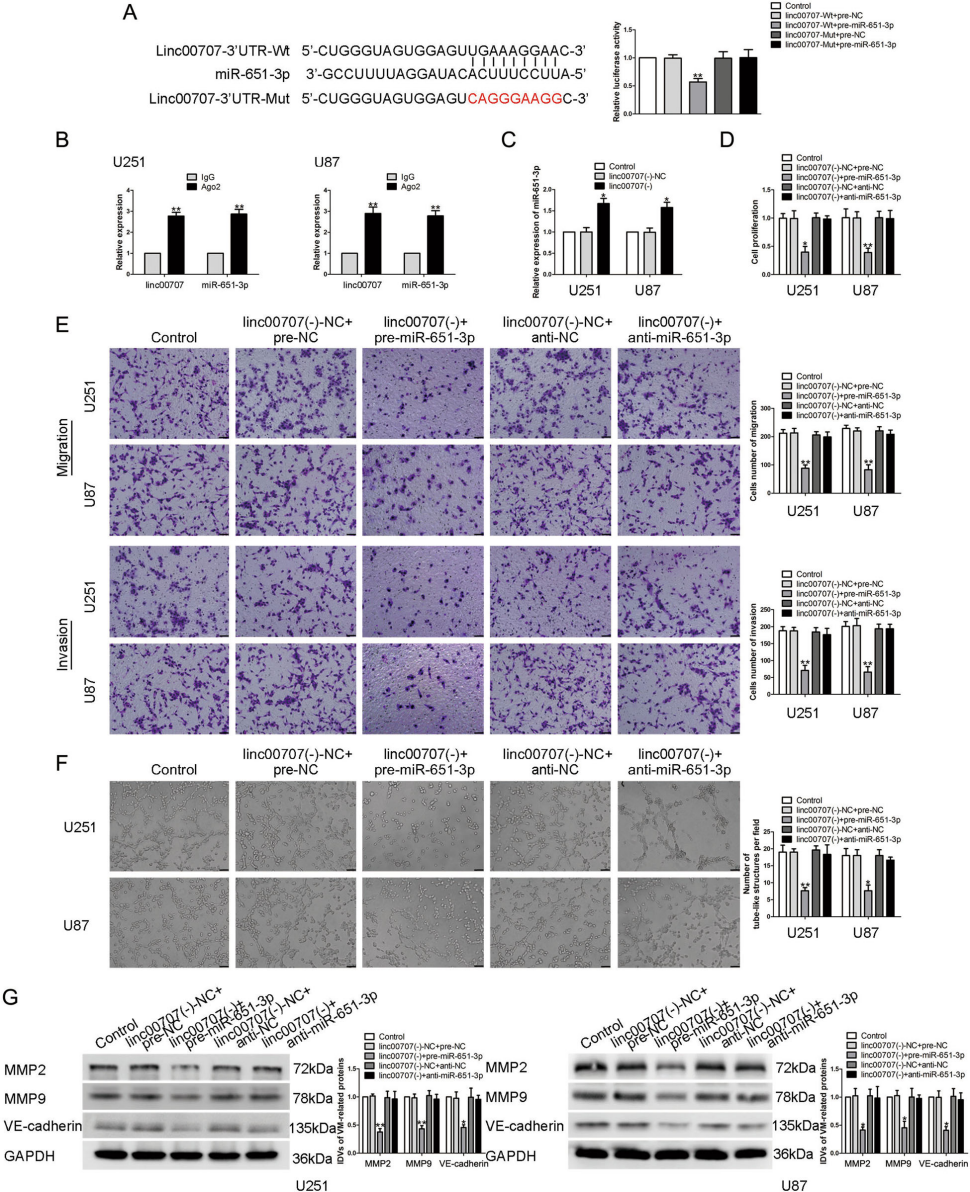
Linc00707 regulated tumor-induced VM via binding to miR-651-3p. **A** The predicted miR-651-3p binding sites in the 3′UTR of linc00707 (linc00707-3′UTR-Wt) and the designed mutant sequence (linc00707-3′UTR-Mut) were indicated. Luciferase reporter assay of HEK-293T cells co-transfected with linc00707-3′UTR-Wt or linc00707-3′UTR-Mut and the indicated miRNA. Data are presented as the mean ± SD (*n* = 3, each group). **P < 0.01 vs. linc00707-Wt + pre-NC group. **B** miR-651-3p was identified in the linc00707-RISC complex. Relative expressions of linc00707 and miR-651-3p were measured using qRT-PCR. Data are presented as the mean ± SD (*n* = 3, each group). **P < 0.01 vs. anti-IgG group. **C** Expression levels of miR-651-3p regulated by linc00707 in U87 and U251 cells. Data are presented as the mean ± SD (*n* = 3, each group). *P < 0.05 vs. linc00707(−)-NC group. **D**–**F** CCK-8 assay, transwell, and three-dimensional culture were applied to evaluate the proliferation, migration, invasion, and tube formation effect of miR-651-3p and linc00707. The scale bar represents 50 µm. **G** The expressions of VM formation-related proteins of U87 and U251 cells after transfected with linc00707 and miR-651-3p plasmid was shown. Data are presented as the mean ± SD (*n* = 3, each group). *P < 0.05, **P < 0.01 vs. linc00707(−)-NC + pre-NC group.

Dual-luciferase reporter assay was conducted to verify the binding effect betwwen linc00707 and miR-651-3p. Compared with linc00707-Wt + pre-NC group, the relative luciferase activity of linc00707-Wt + pre-miR-651-3p group was significantly reduced (Fig. 5A). RIPA showed that compared with anti-IgG group, the enrichment level of linc00707 and miR-651-3p in anti-Ago2 group were significantly increased (Fig. 5B). With the application of qRT-PCR, we found that the expression of miR-651-3p was significantly increased in linc00707(−) group compared with linc00707(−)-NC group (Fig. 5C). We transfected miR-651-3p overexpression and underexpression plasmids into U87 and U251 cells with stably knockdown of linc00707. CCK-8, transwell, and tube formation experiments found that compared with linc00707(−)-NC + pre-NC group, the proliferation, migration, invasion, and the VM formation ability of glioma cells in linc00707(−) + pre-miR-651-3p group was significantly reduced. At the same time, we found that linc00707(−) + anti-miR-651-3p group has no statistically significant difference (Fig. 5D–F). Further application of western blot found that the expressions of MMP2, MMP9, and VE-cadherin in linc00707(−) + pre-miR-651-3p group were significantly decreased compared with linc00707(−)-NC + pre-NC group (Fig. 5G).

### SP2 played a cancer-promoting role in gliomas and bound to the promoter regions of the VM formation-related proteins

**Fig. 6:**
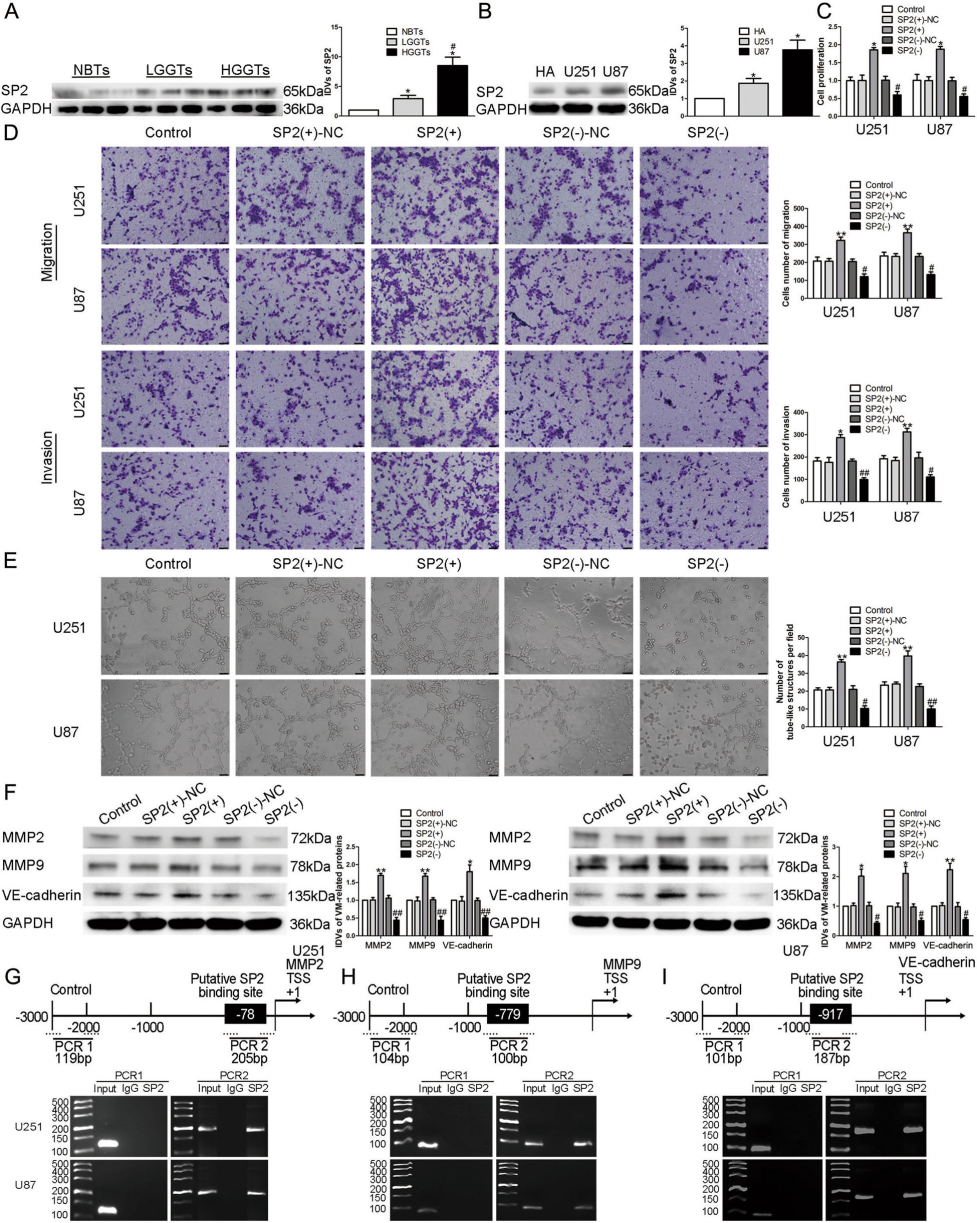
SP2 regulated the expression of VM formation-related proteins at the transcriptional level. **A** Expression levels of SP2 in glioma tissues. Data are presented as the mean ± SD (*n* = 9, each group). *P < 0.05 vs. NBTs group; #*P* < 0.05 vs. LGGTs group. **B** Expression levels of SP2 in glioma cells. Data are presented as the mean ± SD (*n* = 3, each group). *P < 0.05 vs. HA group. **C**–**E** CCK-8 assay, transwell, and three-dimensional culture were applied to evaluate the proliferation, migration, invasion, and tube formation effect of SP2 on U87 and U251 cells. Representative images and corresponding statistical plots are shown. The scale bar represents 50 µm. **F** Protein levels of MMP2, MMP9, and VE-cadherin regulated by SP2 in U87 and U251 cells. Data are presented as the mean ± SD (*n* = 3, each group). *P < 0.05, **P < 0.01 vs. SP2(+)-NC group; #*P* < 0.05, ##*P* < 0.01 vs. SP2(−)-NC group. **G**–**I** Schematic representation of MMP2, MMP9, and VE-cadherin promoter region in 3000 bp upstream of the transcription start site (TSS) designated as +1.Putative SP2 binding sites are shown.

According to the database Targetscan, we found that there maybe a targeted binding site between miR-651-3p and the 3′UTR of SP2. Western blot assay was applied to measure the expression of SP2, we found that SP2 was significantly highly expressed in glioma tissues and cells (Fig. 6A, B). We constructed SP2 overexpressed and knockdown plasmids, and transfected them into U87 and U251 cells to discover its effect. The results revealed that compared with SP2(+)-NC group, glioma cells with overexpressed SP2 showed a significantly improved ability in proliferation, migration, invasion, and VM formation, while SP2(−) group was significantly reduced compared to SP2(−)-NC group (Fig. 6C–E). qRT-PCR and western blot showed that in SP2(+) group, the expressions of MMP2, MMP9, and VE-cadherin were significantly increased, while the SP2(−) group showed an opposite effect (Fig. 6F and Supplementary Fig. 6A). Due to the Jaspar database, we discovered that there might be binding sites between SP2 and the promoter regions of the VM formation-related proteins. The binding effects were verified by ChIP assay, proved that SP2 regulated the expression of MMP2, MMP9, and VE-cadherin at the transcription level (Fig. 6G–I).

### miR-651-3p targeted and negatively regulate SP2 expression

**Fig. 7:**
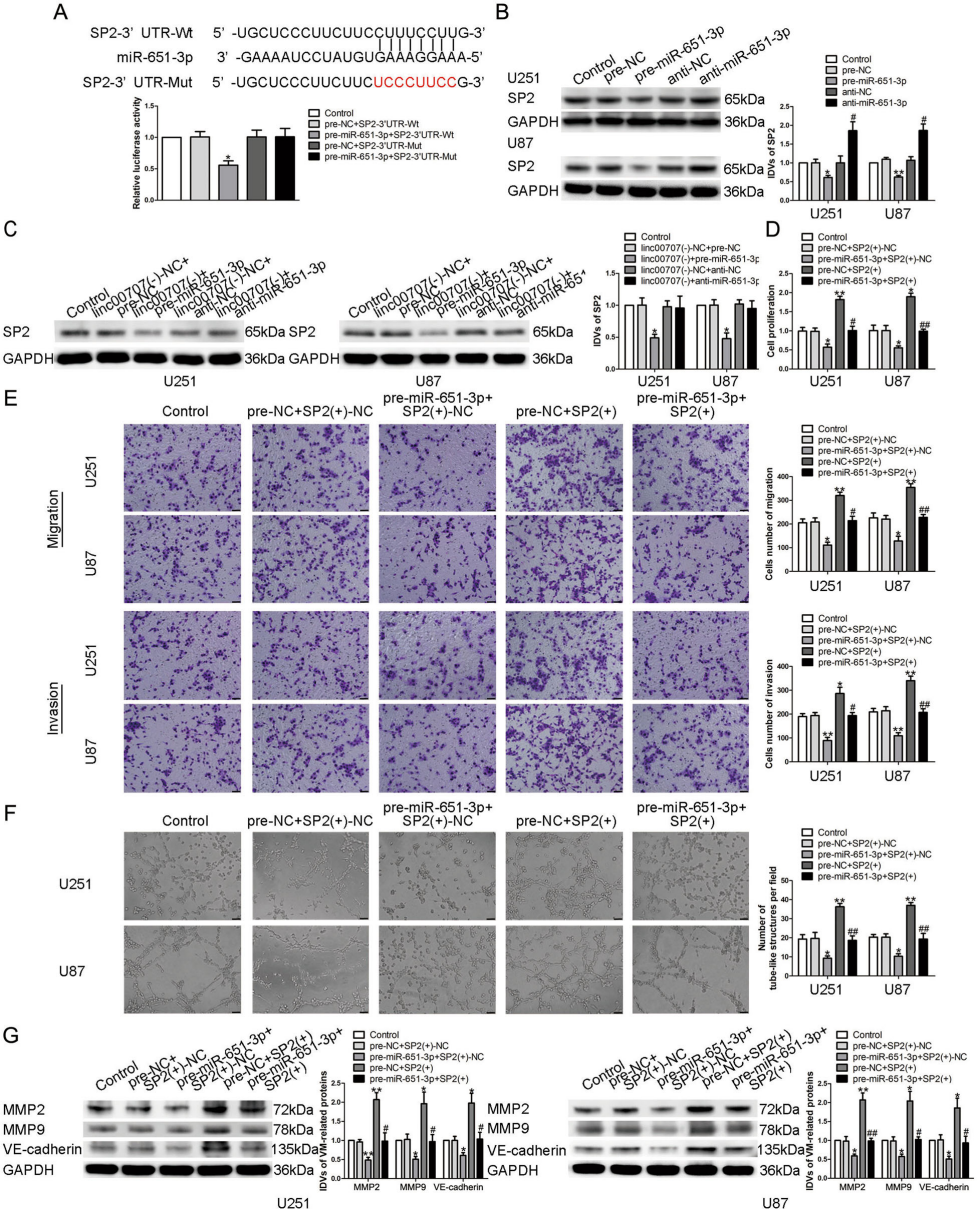
miR-651-3p regulated tumor-induced VM formation via binding to SP2. **A** The predicted miR-651-3p binding sites in the 3′UTR of SP2 (SP2-3′UTR-Wt) and the designed mutant sequence (SP2-3′UTR-Mut) were indicated. Luciferase reporter assay of HEK-293T cells co-transfected with SP2-3′UTR-Wt or the SP2-3′UTR-Mut and the indicated miRNA. Data are presented as the mean ± SD (*n* = 3, each group). *P < 0.05 vs. pre-NC + SP2-3′UTR-Wt group. **B** Expression levels of SP2 regulated by miR-651-3p in U87 and U251 cells. Data are presented as the mean ± SD (*n* = 3, each group). *P < 0.05, **P < 0.01 vs. pre-NC group; #*P* < 0.05 vs. anti-NC group. **C** The expression of SP2 in U87 and U251 cells after co-transfection with linc00707 and miR-651-3p plasmid was shown. Data are presented as the mean ± SD (*n* = 3, each group). *P < 0.05 vs. linc00707(−)-NC + pre-NC group. **D**–**F** CCK-8 assay, transwell, and three-dimensional culture were applied to evaluate the proliferation, migration, invasion, and tube formation effect of miR-651-3p and SP2. The scale bar represents 50 µm. **G** The expression of VM formation-related proteins in U87 and U251 cells after co-transfection with miR-651-3p and SP2 plasmid was shown. Data are presented as the mean ± SD (*n* = 3, each group). *P < 0.05,**P < 0.01 vs. pre-NC + SP2(+)-NC group; #*P* < 0.05, ##*P* < 0.01 vs. pre-NC + SP2(+) group.

Dual-luciferase reporter gene analysis showed that the relative luciferase activity of pre-miR-651-3p + SP2-3′UTR-Wt group was significantly reduced compared with pre-NC + SP2-3′UTR-Wt group. However, there was no significant difference in pre-miR-651-3p + SP2-3′UTR-Mut group (Fig. 7A). qRT-PCR and western blot results showed that compared with pre-NC group, SP2 expression in pre-miR-651-3p group was significantly decreased, but significantly increased in anti-miR-651-3p group (Fig. 7B and Supplementary Fig. 5A). Meanwhile, we found that SP2 expression in linc00707(−) + pre-miR-651-3p group was significantly reduced compared with linc00707(−)-NC + pre-NC group (Fig. 7C and Supplementary Fig. 5B). In U87 and U251 cells with stably overexpressed SP2, overexpression and knockdown plasmids of miR-651-3p were transfected to detect their effects. We found that in pre-miR-651-3p + SP2(+)-NC group, the proliferation, migration, invasion, and VM formation ability of glioma cells were significantly reduced, while they were significantly increased in pre-NC + SP2(+) group. Compared with pre-NC + SP2(+) group, those abilities of glioma cells were significantly reduced in the pre-miR-651-3p + SP2(+) group (Fig. 7D–F). qRT-PCR and western blot was used to detect changes in the expressions of VM formation-related proteins. We found that compared with the control group, the expressions of the proteins in pre-miR-651-3p + SP2(+)-NC group were significantly reduced, while the expressions of the above proteins in pre-NC + SP2(+) group were significantly increased (Fig. 7G and Supplementary Fig. 6B).

### Knockdown of HNRNPD and linc00707 combination with overexpressed ZHX2 suppressed tumor growth and induced higher survival period in nude mice

**Fig. 8:**
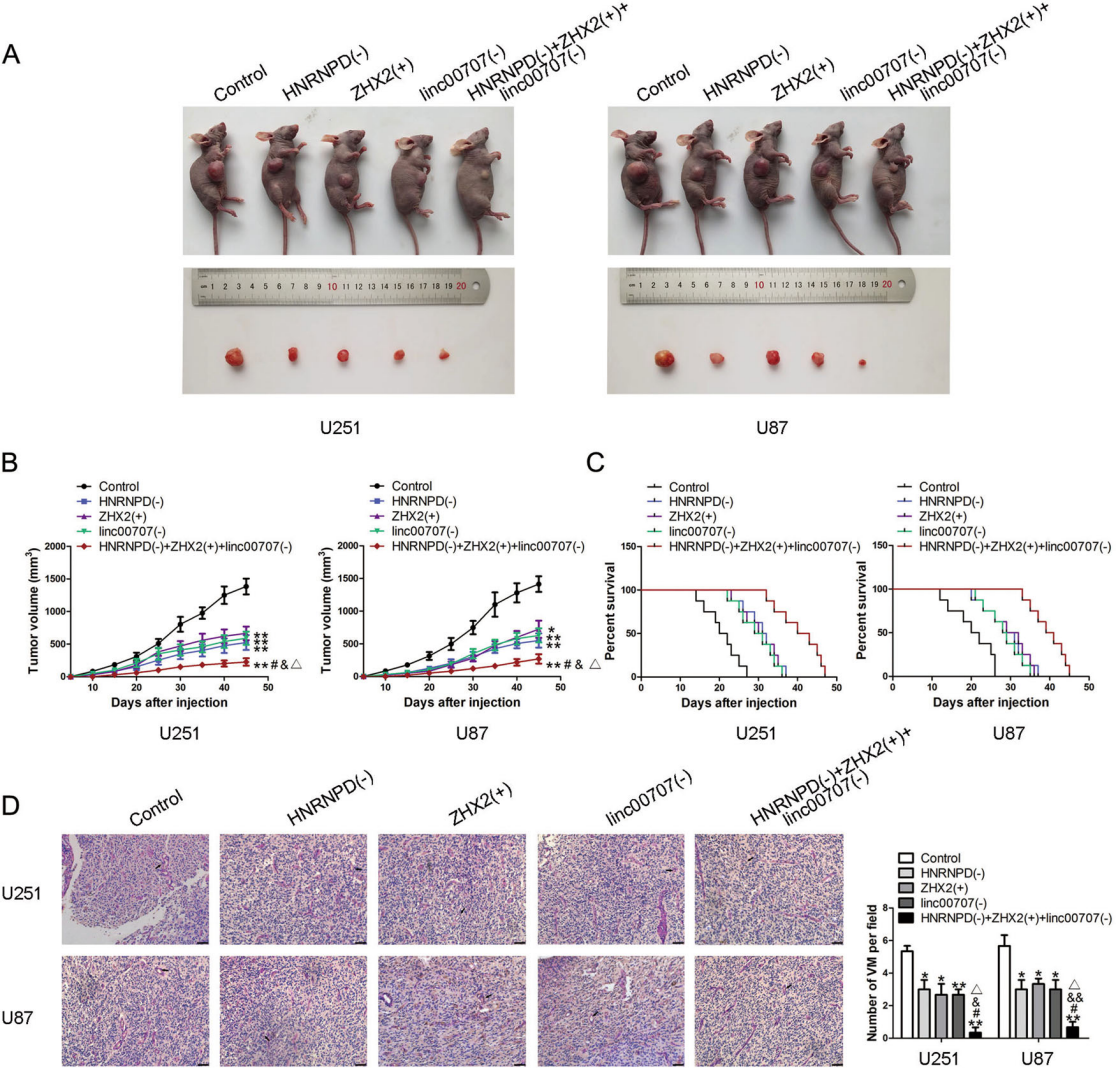
Tumor xenograft studies. **A** The nude mice carried tumors from respective groups were shown. The sample tumors from respective groups were shown. **B** Tumor growth curves were shown. *P < 0.05, **P < 0.01 vs. control group; #*P* < 0.05 vs. HNRNPD(−) group; &*P* < 0.05 vs. ZHX2(+) group; △*P* < 0.05 vs. linc00707(−) group. Using one-way analysis of variance for statistical analysis. **C** Survival curves from nude mice were shown (*n* = 8, each group). **D** CD31-PAS staining was used to detect the VM structures in xenografted tumor. Data are presented as mean ± SD (*n* = 8, each group). *P < 0.05,**P < 0.01 vs. control group; #*P* < 0.05 vs. HNRNPD(−) group; &*P* < 0.05, &&*P* < 0.01 vs. ZHX2(+) group; △*P* < 0.05 vs. linc00707(−) group. The scale bar represents 50 µm. The arrow indicates the VM structure.

The nude mouse xenograft model was used to further study the effects of HNRNPD, ZHX2, and linc00707 on the occurrence and development of glioma. Nude mice are divided into five groups as shown. Approximately 6 × 10^5^ tumor cells were injected into the right armpit of nude mice and after 45 days, we found that compared with the control group, the transplanted tumor volume of nude mice was significantly reduced in HNRNPD(−) group, ZHX2(+) group, linc0007(−) group, and HNRNPD(−) + ZHX2(+) + linc0007(−) group. Moreover, HNRNPD(−) + ZHX2(+) + linc00707(−) group produced the smallest volume of the transplanted tumors (Fig. 8A, B). The survival analysis showed that HNRNPD(−), ZHX2(+), and linc0007(−) groups exhibited a longer survival period compared with the control group. Moreover, the HNRNPD(−) + ZHX2(+) + linc0007(−) group showed the longest survival period (Fig. 8C). As shown in Fig. 8D, the HNRNPD(−) + ZHX2(+) + linc0007(−) group showed the lowest density of VM structures among all the groups.

## Discussion

Our study confirmed for the first time that HNRNPD and linc00707 are highly expressed in glioma tissues and cells, while ZHX2 and miR-651-3p are low expressed. Knockdown of HNRNPD, linc00707, and overexpression of ZHX2, miR-651-3p significantly inhibit the VM formation ability of glioma cells.

The mechanisms of RBPs regulating tumorigenesis and tumor development have attracted more and more attention recent years^28^. Our study found that HNRNPD was highly expressed in glioma tissues and cells, knockdown of HNRNPD significantly reduced the expression of MMP2, MMP9, and VE-cadherin in U87 and U251 cells, further inhibited the VM formation. Other studies have also shown the important function of RBPs in glioma and VM formation. RBP-PTBP1 is highly expressed in glioma tissues and cells, and activate the expression of ADAR1 transcript through internal ribosome entry site, which plays an important role in the occurrence and development of glioma^29^. RBP-ZRANB2 is overexpressed in glioma cells, knockdown of ZRANB2 inhibits the ability of VM formation in glioma cells through SNHG20/FOXK1 pathway^30^.

ZHX2 is involved in regulating protein hydroxylation, lipid balance, DNA methylation, cell proliferation, and the occurrence and development of various tumors^11,31,32^. Our study demonstrated that ZHX2 was underexpressed in glioma tissues and cells. Overexpression of ZHX2 significantly suppressed the VM forming ability of glioma cells. Other studies also discovered that ZHX2 is underexpressed in hepatocellular carcinoma tissues and cells and exerted a transcriptional repression role. Overexpression of ZHX2 promotes the expression of miR-155, which in turn inhibits the growth of hepatocellular carcinoma cells^33,34^.

Researches have shown that RBPs bound with RNAs could play a variety of biological regulatory roles. Some members of the HNRNP family can regulate the occurrence and development of tumors by regulating the stability of mRNA, which is rich in AU elements in its 3′UTR. For example, HNRNPD inhibits the expression of c-Myc mRNA by decreasing its stability, thereby suppresses the growth of bladder cancer cells^35^. HNRNPF increases the stability of Snail1 mRNA and promotes the epithelial–mesenchymal transition of bladder cancer cells^36^. In colon cancer, HNRNPD reduces ATX mRNA stability by recognizing the AU element in its 3′UTR and further promotes the migration of colon cancer cells^37^. In our study, based on the bioinformatics database AREsite2, AU elements were found in the 3′UTR of ZHX2 mRNA. The dual-luciferase reporter assay was then used to verify the binding between HNRNPD and the AU elements. Interestingly, our experiment also revealed that knockdown of HNRNPD could significantly prolong the half-life of ZHX2 mRNA. Meanwhile, we discovered that compared with knockdown of HNRNPD or overexpression of ZHX2 alone, the combination of the two could significantly inhibit the VM formation of glioma cells. The above results indicated that knockdown of HNRNPD could inhibit the VM formation ability of glioma cells through increasing the stability of ZHX2 mRNA.

Studies have shown that lncRNAs play an important part in regulating the biological behaviors of tumor cells^38^. Our study demonstrated that linc00707 is highly expressed in glioma tissues and cells. Knockdown of linc00707 significantly inhibited the VM formation ability of U87 and U251 cells. This result is in accordance with the role of linc00707 in other tumors. For instance, linc00707 promotes the development of hepatocellular carcinoma by activating ERK/JNK/AKT pathway^39^. Knockdown of linc00707 inhibits the proliferation and migration of lung adenocarcinoma cells and promotes cell apoptosis^40^. Other lncRNAs are also involved in the VM formation of tumor cells. LncRNA HOXA-AS2 binds to miR-373 and negatively regulates its expression, followed by promoting the VM formation ability of glioma cells^41^. LncRNA MALAT1 vias VE-cadherin/β-catenin complex, ERK/MMP, and FAK/paxillin signal transduction pathway to regulate the VM formation of gastric cancer cells^42^.

Studies have shown that transcription factors can specifically bind to the promoter region of lncRNAs, regulates their expression, and further regulates the occurrence and development of tumors. SP1 specifically binds to the promoter region of linc00313, plays a role in promoting transcription, and enhances the proliferation, migration, and invasion ability of papillary thyroid cancer cells^43^. Transcription factor IRF1 binds to the promoter region of lncRNA GAS5, promotes the occurrence and development of osteosarcoma^44^. Transcription factor YY1 plays a role in promoting transcription by combining with the promoter region of lncRNA PVT1, increases the proliferation of lung cancer cells, and inhibites their apoptosis^45^. Based on the bioinformatics database Jaspar, we found a potential binding site between ZHX2 and the promoter region of linc00707, further application of the ChIP assay confirmed this combination, suggested that ZHX2 regulated the expression of linc00707 at the transcription level.

Many miRNAs are abnormally expressed in tumors and participate in regulating the biological behavior and VM formation of tumor cells. Previous studies have shown that overexpression of miR-9, miR-584-3p, and miR-141 can significantly inhibit the VM formation in glioma cells^46–48^. Our study has found that miR-651-3p was underexpressed in glioma tissues and cells. Overexpression of miR-651-3p significantly inhibited the VM formation. We further analyzed the bioinformatics database DIANA, and found that there maybe a binding site between linc00707 and miR-651-3p. The dual-luciferase reporter assay confirmed the binding effect. Further, RIP experiments confirmed that linc00707 and miR-651-3p were in the same RISC complex. In U87 and U251 cells, knockdown of linc00707 significantly increased the expression of miR-651-3p. Meanwhile, we found that knockdown of both linc00707 and miR-651-3p could reverse the miR-651-3p repression function on its target gene SP2 and the ability of VM formation in glioma cells. A large number of studies have shown that lncRNAs can act as ceRNA through binding with miRNA, in order to inhibit the negative regulation of miRNA on its target genes and further regulate the development of tumors. For example, lncRNA DANCR inhibits the negative regulation of miR-634 on its downstream target gene RAB1A by binding to miR-634, and promotes the development of gliomas^49^. Linc00707 binds to miR-206 and inhibits its negative regulation against NOTCH3 and TM4SF1, promotes the proliferation and metastasis of colorectal cancer cells^50^. Linc00339 inhibits the negative regulation of miR-539-5p on TWIST1 by competitively binding with miR-539-5p, enhances the ability of VM formation in glioma cells^51^.

SP2 is involved in regulating cells proliferation, metabolism, neural development, cell signaling, and the occurrence and development of various tumors^52–54^. Previous studies have found that transgenic mice with overexpressed SP2 have increased sensitivity to wound and carcinogen-induced mastoid formation in epidermal basal keratinocytes^22^. Our study found that SP2 is highly expressed in glioma tissues and cells, knockdown of SP2 significantly inhibited the VM formation ability. Based on the analysis of the bioinformatics database Targetscan, we found that there existed a potential binding site between miR-651-3p and the 3′UTR of SP2 mRNA, dual-luciferase reporter assay was used to confirmed the combination. We also found that overexpression of miR-651-3p significantly inhibited the expression of SP2. Meanwhile, overexpression of SP2 could reverse the negative effect of miR-651-3p on glioma cells. It is suggested that miR-651-3p binds to the 3′UTR of SP2 mRNA and negatively regulate its expression, thereby regulates the formation of VM in glioma cells. Studies have shown that miRNAs can target the 3′UTR of their downstream transcription factor mRNAs and participate in regulating the biological behavior of tumor cells. For instance, miR-638 binds with SP2 mRNA 3′UTR and negatively regulate SP2 expression, therefore inhibits the proliferation of gastric cancer cells^21^. miR-876-5p targets WNT5A and MITF mRNA 3′UTR, represses the proliferation and migration ability of gastric cancer cells^55^. miR-141 binds to the 3′UTR of its downstream target gene ZEB1, suppresses the VM formation ability of breast cancer cells^56^.

MMP2, MMP9, and VE-cadherin are classic proteins associated with VM formation in glioma cells, and their high expressions suggest enhanced VM formation ability^57–59^. In this study, based on the bioinformatics database Jaspar, SP2 was found to has potential binding sites with the promoter regions of MMP2, MMP9, and VE-cadherin. Further application of ChIP assay verified the combination. Knockdown of SP2 significantly inhibited the mRNA and protein expression level of MMP2, MMP9, and VE-cadherin, thereby repressed the VM formation ability of glioma cells. Another research has also found similar function of SP2. It increases the formation of blood vessels of clear renal cell sarcoma cells by promoting the transcription of VEGFA^60^. It is indicated that SP2 can regulate the expression of MMP2, MMP9, and VE-cadherin and promote the formation of VM in glioma cells at the transcriptional level.

Finally, xenograft tumor experiments in nude mice showed that compared with the control group, the volume of xenograft tumors in HNRNPD(−), ZHX2(+), linc00707(−) group, as well as the combination of the three were significantly decreased and associated with longer survival period. Moreover, the combination of the three has the smallest size of the transplanted tumor and the longest survival period, indicated that the combination of the three has the best effect.

In summary, our study confirmed that knockdown of HNRNPD increased the stability of ZHX2 mRNA, then the upregulated ZHX2 increased its transcriptional repression effect on linc00707, the downregulated linc00707 reduced its binding with miR-651-3p, and increased the negative regulation of miR-651-3p on its target gene SP2. In response, the downregulated SP2 inhibits the transcription of VM formation-related proteins MMP2, MMP9, and VE-cadherin, thereby inhibited the VM formation ability of U87 and U251 cells. The results of the study may provide new ideas for the anti-VM therapy of gliomas.

## Supplementary information

Supplementary material 1

Supplementary material 2

Supplementary material 3

Supplementary material 4

Supplementary material 5

Supplementary material 6

Supplementary material 7

Supplementary material 8

Supplementary Table 1

Supplementary Table 2

Supplementary Table 3
