# Supplementary figures and images for: HNRNPD interacts with ZHX2 regulating the vasculogenic mimicry formation of glioma cells via linc00707/miR-651-3p/SP2 axis
Source: Cell Death Dis. 2021 Feb 4;12(2):153. doi: 10.1038/s41419-021-03432-1 (PMC7862279; doi:10.1038/s41419-021-03432-1)

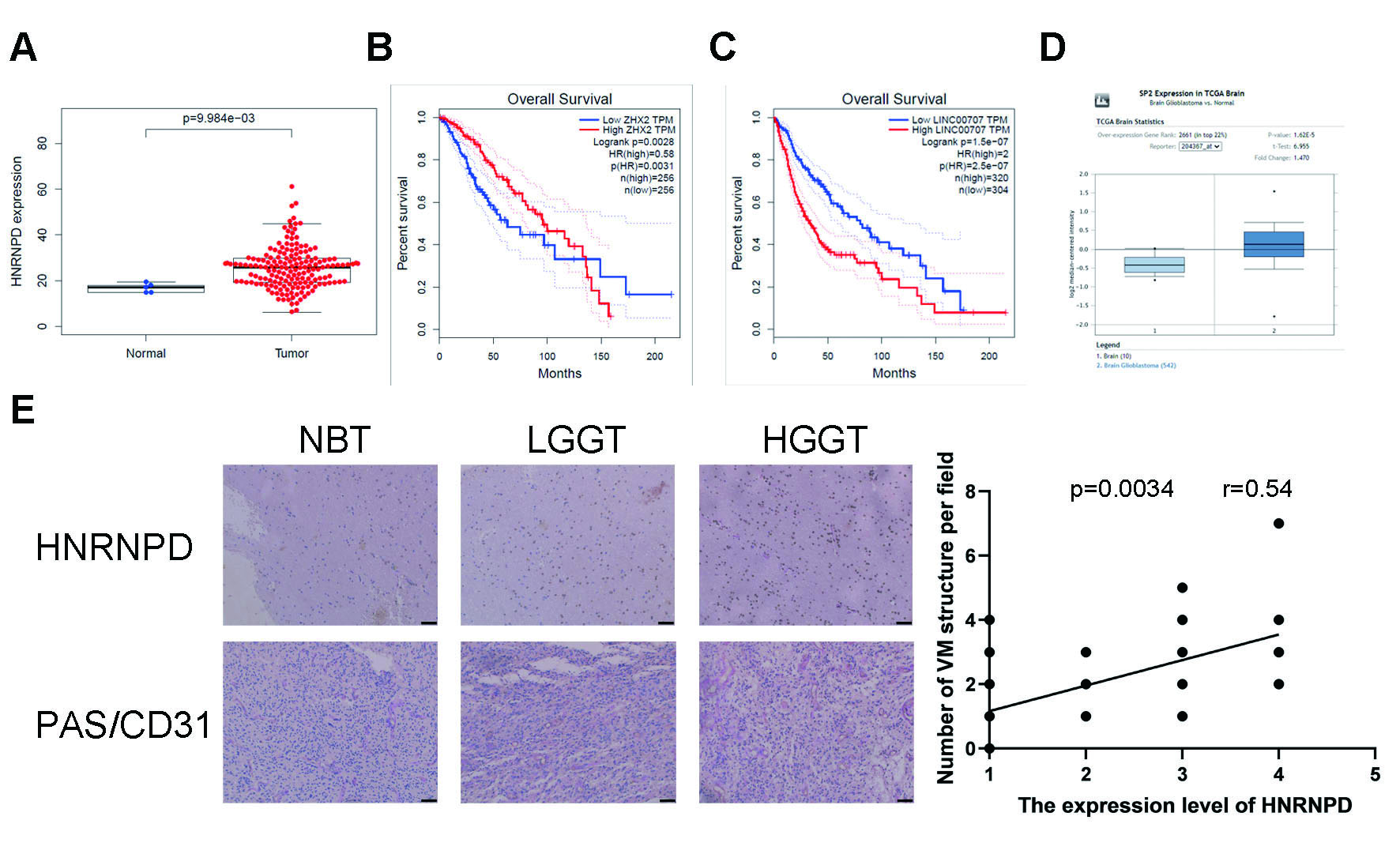

Supplement: Supplementary file 1 — Supplementary material 1 [file 41419_2021_3432_MOESM1_ESM.jpg]

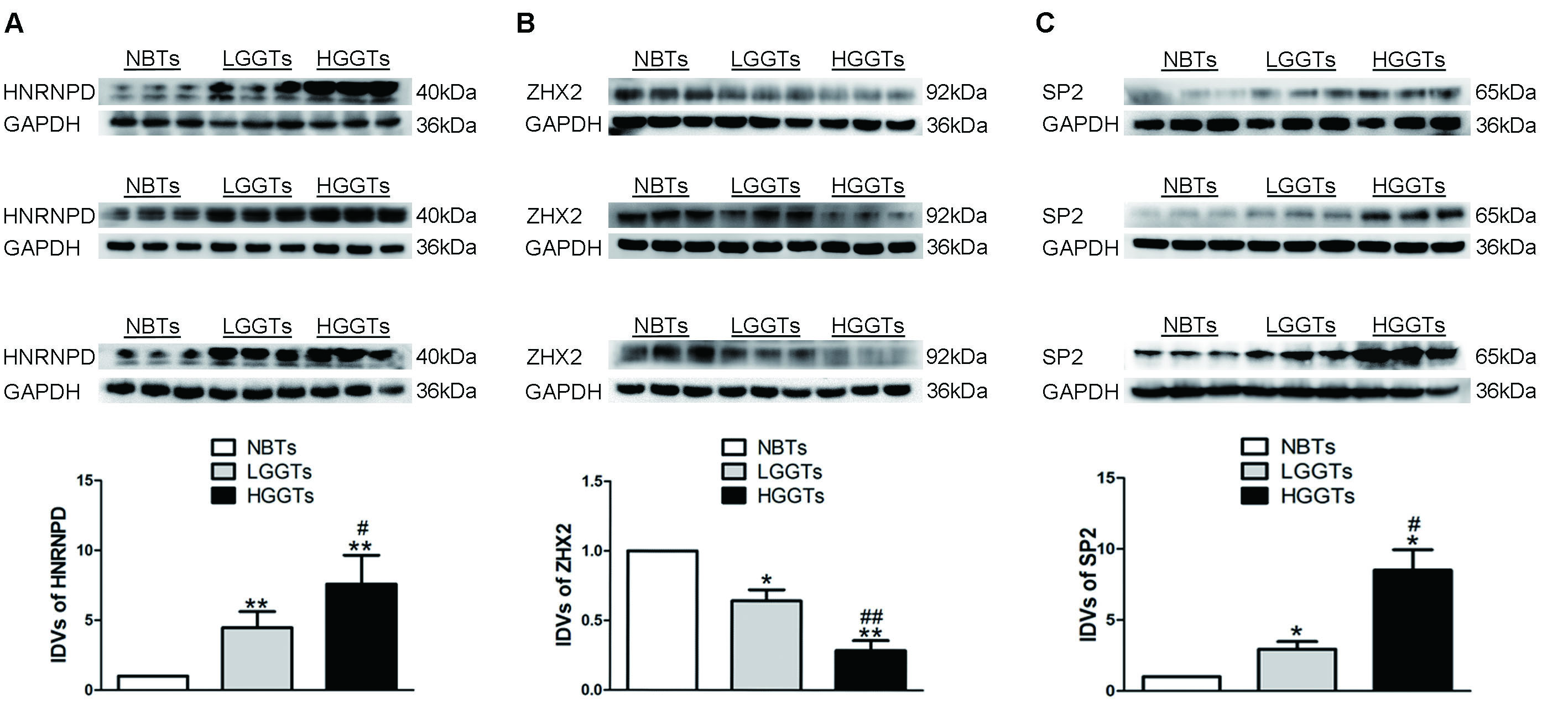

Supplement: Supplementary file 2 — Supplementary material 2 [file 41419_2021_3432_MOESM2_ESM.jpg]

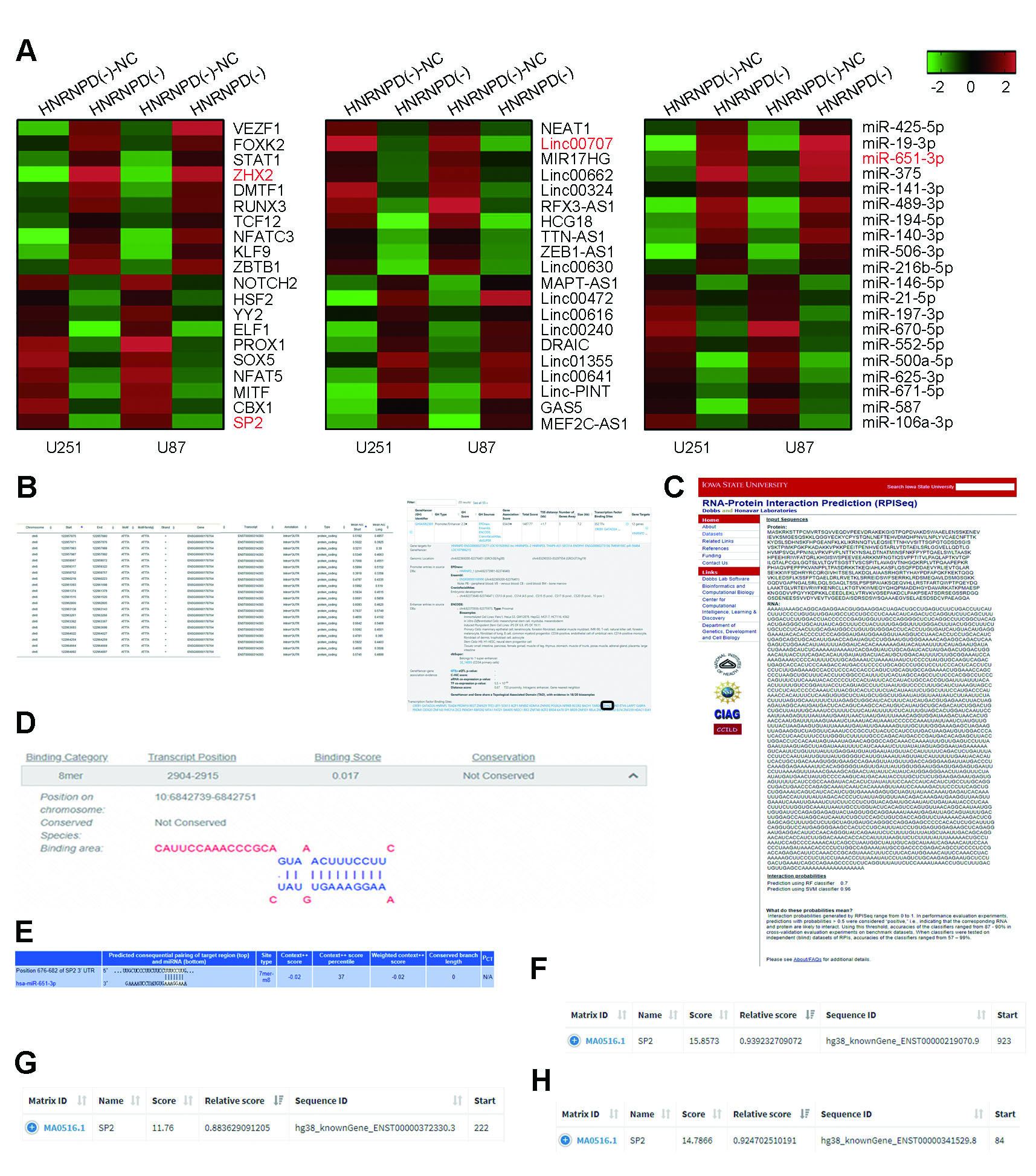

Supplement: Supplementary file 3 — Supplementary material 3 [file 41419_2021_3432_MOESM3_ESM.jpg]

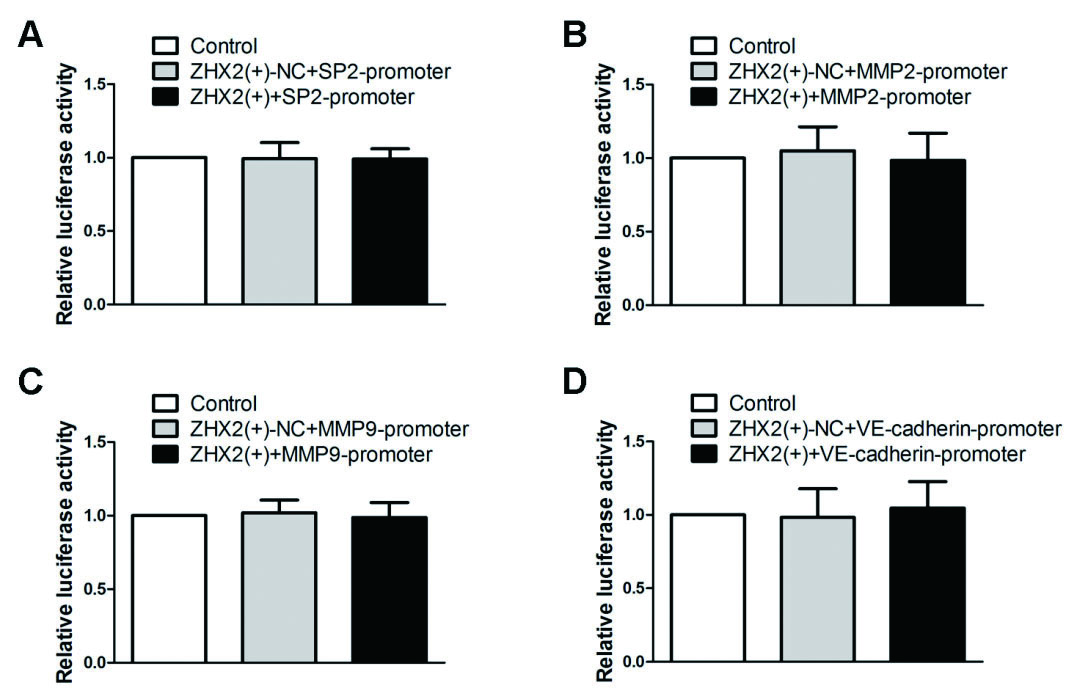

Supplement: Supplementary file 4 — Supplementary material 4 [file 41419_2021_3432_MOESM4_ESM.jpg]

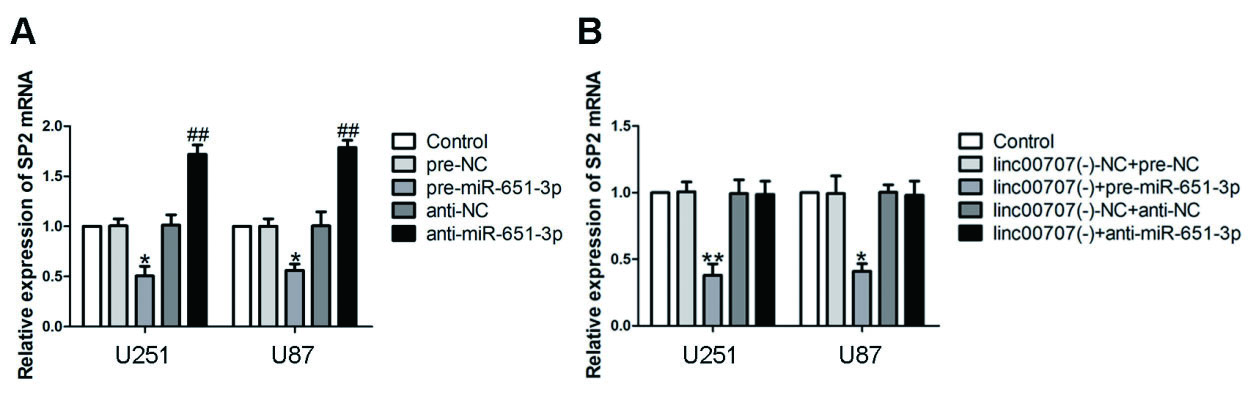

Supplement: Supplementary file 5 — Supplementary material 5 [file 41419_2021_3432_MOESM5_ESM.jpg]

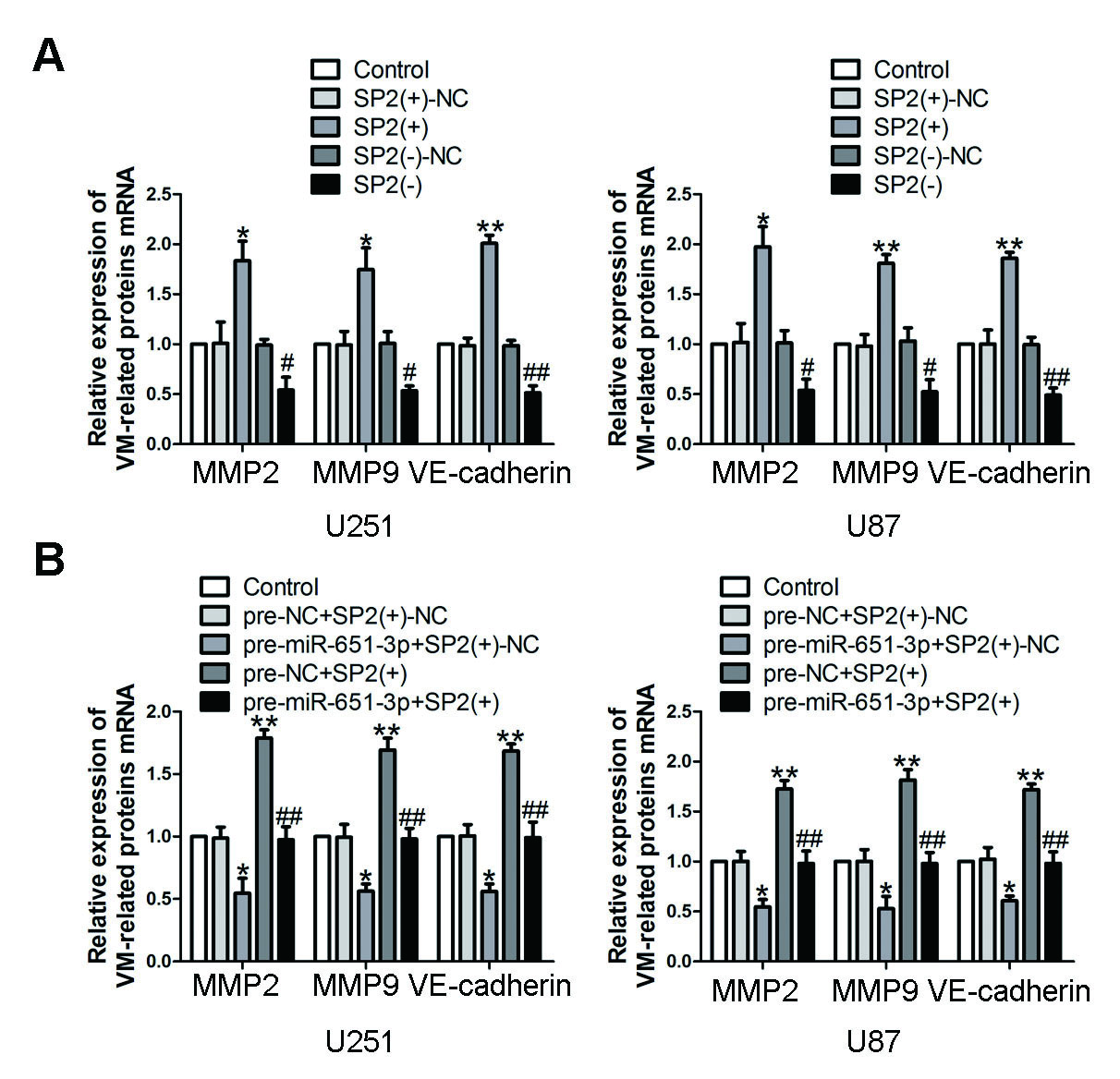

Supplement: Supplementary file 6 — Supplementary material 6 [file 41419_2021_3432_MOESM6_ESM.jpg]

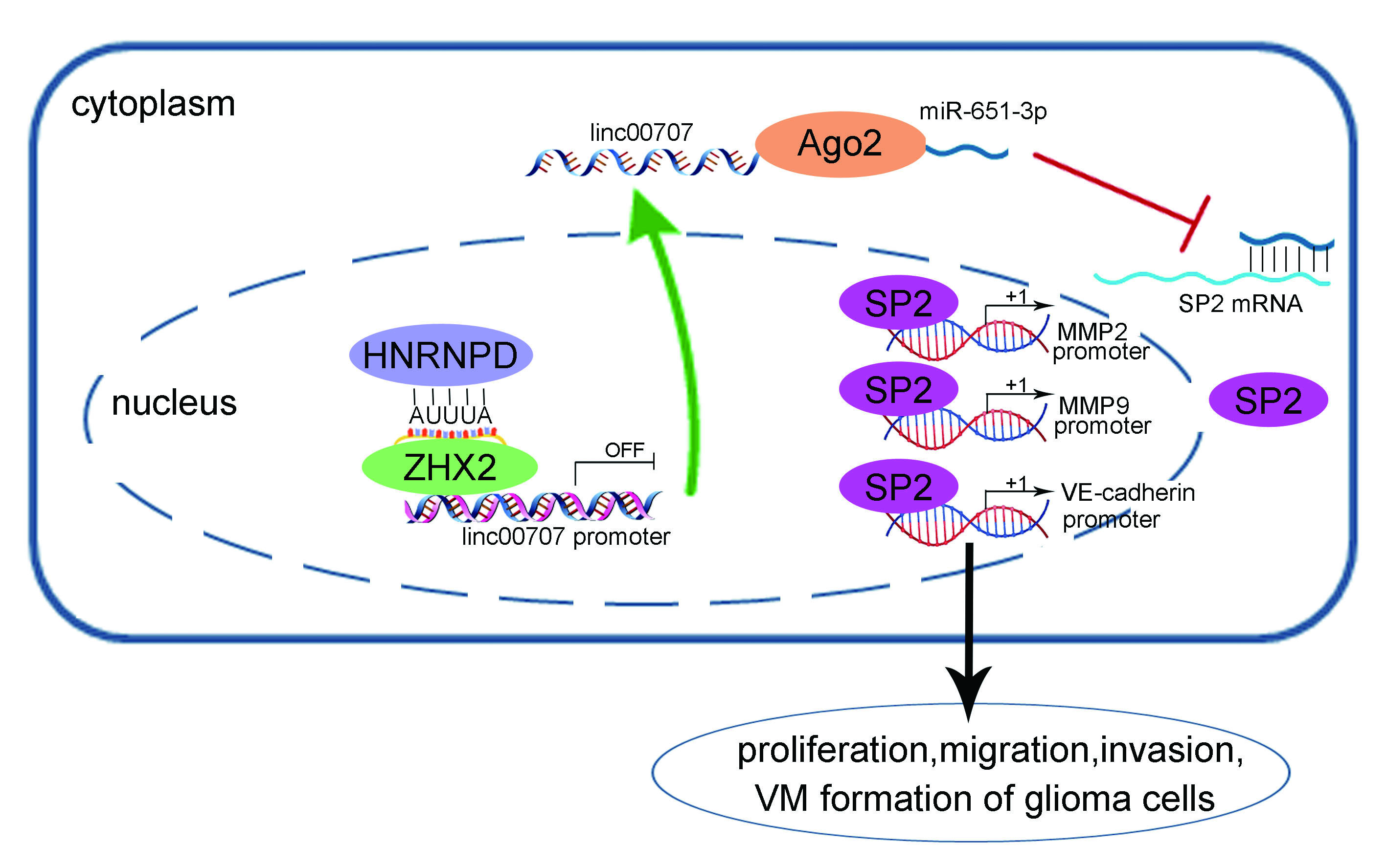

Supplement: Supplementary file 7 — Supplementary material 7 [file 41419_2021_3432_MOESM7_ESM.jpg]
