# Supplementary material for: HNRNPD interacts with ZHX2 regulating the vasculogenic mimicry formation of glioma cells via linc00707/miR-651-3p/SP2 axis
Source: Cell Death Dis. 2021 Feb 4;12(2):153. doi: 10.1038/s41419-021-03432-1 (PMC7862279; doi:10.1038/s41419-021-03432-1)
Supplement: Supplementary file 8 — Supplementary material 8 [file 41419_2021_3432_MOESM8_ESM.docx]

**Supplementary information**

**Supplementary material 1**

Supplementary Figure 1. Bioinformatics database prediction results.

1. The predicted expression of HNRNPD in glioma using database TCGA. (B)-(C) The predicted overall survival of ZHX2 and linc00707 in glioma using database GEPIA. (D) The predicted expression of SP2 in glioma using database Oncomine. (E) The Spearman correlation analysis between HNRNPD and VM formation.

**Supplementary material 2**

Supplementary Figure 2. The original data of the Western Blot assay for the endogenous expression of HNRNPD, ZHX2 and SP2 in glioma tissues.

**Supplementary material 3**

Supplementary Figure 3. (A) The microarray analysis of total RNAs isolated from HNRNPD(-)-NC and HNRNPD(-) cells. Red indicates high relative expression and green indicates low relative expression. (B)-(H) The predicted binding effects and binding sites.

**Supplementary material 4**

Supplementary Figure 4. The relative luciferase activtity of SP2, MMP2, MMP9 and VE-cadherin promoters after co-transfected with ZHX2 Data are presented as the mean±SD (n=3, each group). *P*>0.05 vs. ZHX2(+)-NC+SP2/MMP2/MMP9/VE-cadherin promoter group.

**Supplementary material 5**

Supplementary Figure 5. SP2 mRNA expression regulated by miR-651-3p and linc00707.

1. qRT-PCR was used to measure SP2 expression after miR-651-3p overexpression or knockdown. Data are presented as the mean±SD (n=3, each group). **P*<0.05 vs. pre-NC group; ##*P*<0.01 vs. anti-NC group. (B) qRT-PCR was used to measure SP2 expression regulated by linc00707 and miR-651-3p. Data are presented as the mean±SD (n=3, each group). **P* <0.05,***P*<0.01 vs. sh-NC+pre-NC group.

**Supplementary material 6**

Supplementary Figure 6. The mRNA levels of the VM formation-related proteins regulated by miR-651-3p and SP2.

1. mRNA levels of MMP2, MMP9 and VE-cadherin regulated by SP2 in U87 and U251 cells. Data are presented as the mean±SD (n=3, each group). **P*<0.05,***P*<0.01 vs. SP2(+)-NC group; #*P*<0.05,##*P*<0.01 vs. SP2(-)-NC group. (B) mRNA levels of MMP2, MMP9 and VE-cadherin regulated by miR-651-3p and SP2. Data are presented as the mean±SD (n=3, each group). **P*<0.05,***P*<0.01 vs. pre-NC+ SP2(+)-NC group; ##*P*<0.01 vs. pre-NC+SP2(+) group.

**Supplementary material 7**

Supplementary Figure 7. The schematic diagram of the study.
