# Supplementary material for: HNRNPD interacts with ZHX2 regulating the vasculogenic mimicry formation of glioma cells via linc00707/miR-651-3p/SP2 axis
Source: Cell Death Dis. 2021 Feb 4;12(2):153. doi: 10.1038/s41419-021-03432-1 (PMC7862279; doi:10.1038/s41419-021-03432-1)
Supplement: Supplementary file 9 — Supplementary Table 1 [file 41419_2021_3432_MOESM9_ESM.docx]

STable 1: Primer list of PCR

| Gene name | Forward | Reverse |
| --- | --- | --- |
| GAPDH | GGAAGCTTGTCATCAATGGAAATC | TGATGACCCTTTTGGCTCCC |
| HNRNPD | TTTTGGTGAGGTGGAATCCATA | GATATTGTTCCTTCGACATGGC |
| ZHX2 | AGAAAACACAGGGTCAGGTTAA | CTGGGCAGAGAACTTGTTAACT |
| Linc00707 | TCACATCTGTGAAAAGAGTGCT | CTGGACTGTGAGTACCAGGC |
| MMP2 | ATTGTATTTGATGGCATCGCTC | ATTCATTCCCTGCAAAGAACAC |
| MMP9 | CAGTACCGAGAGAAAGCCTATT | CAGGATGTCATAGGTCACGTAG |
| VE-cadherin | AAAGAATCCATTGTGCAAGTCC | CGTGTTATCGTGATTATCCGTG |
