# Supplementary material for: HNRNPD interacts with ZHX2 regulating the vasculogenic mimicry formation of glioma cells via linc00707/miR-651-3p/SP2 axis
Source: Cell Death Dis. 2021 Feb 4;12(2):153. doi: 10.1038/s41419-021-03432-1 (PMC7862279; doi:10.1038/s41419-021-03432-1)
Supplement: Supplementary file 10 — Supplementary Table 2 [file 41419_2021_3432_MOESM10_ESM.docx]

STable 2: Sequence of the applied plasmid

| Gene name | Sequence |
| --- | --- |
| sh-HNRNPD | GCTGGGACACTACAAAGAA |
| sh-linc00707 | GCACTGGATCAATTCCAATTA |
| sh-SP2 | GGACCCGATCAAATGCCAATA |
