# Supplementary material for: HNRNPD interacts with ZHX2 regulating the vasculogenic mimicry formation of glioma cells via linc00707/miR-651-3p/SP2 axis
Source: Cell Death Dis. 2021 Feb 4;12(2):153. doi: 10.1038/s41419-021-03432-1 (PMC7862279; doi:10.1038/s41419-021-03432-1)
Supplement: Supplementary file 11 — Supplementary Table 3 [file 41419_2021_3432_MOESM11_ESM.docx]

STable 3: Primer sequence of ChIP

| Gene name | Forward | Reverse | Product size |
| --- | --- | --- | --- |
| Control | GGCTGCATTATCAGCTTTCC | TCAGATGTGCAGGAGGTGTT | 177bp |
| Linc00707 | TACTTCGTGCCAGGTGTGGT | TCCCTCTGAAAAGAACATGG | 150bp |
|  |  |  |  |
| Control | AGCACATGCAATACCTGAACA | ATCACCTGTCTGGGGCAGT | 119bp |
| MMP2 | GGGGAAAAGAGGTGGAGAAA | GGGACCCTGGAAAAATCACT | 205bp |
|  |  |  |  |
| Control | TACACTCGGGTGGCAGAGAT | CTATCCAGCTCACCGGTCTC | 104bp |
| MMP9 | AATCCTCACATCAATTTAGGGACA | GACCTCGGGCAAATGTCTTA | 100bp |
|  |  |  |  |
| Control | CGCCAAAAGAGAGATTGGAT | CGACTCACGCTTGACTTGAT | 101bp |
| VE-cadherin | GGTGAGCCCAGTCCTCTGTA | ACAGCCTCATCCTCACAAGG | 187bp |
